# The Relief of Distress

**Published:** 1904-10-29

**Authors:** 


### The Hospital.

A Journal of

The Medical Sciences and Hospital Administration.

Vol. XXXVII—No. 944. OCTOBER 29, 1904.

### The Relief of Distress.

The relief of distress, when it is real and resulting from temporary causes, is so intimately associated with the preservation of the public health as to call for the cordial co-operation of all sanitarians in any endeavour to accomplish it in the best and most effective way; and we have therefore no hesitation in calling the attention of our readers to the improved prospects, in this direction, which have been opened out by the sagacity of the President of the Local Government Board. Mr. Long is, we think, the first holder of that office who has at all realised its possibilities as an agency for the control of disease; and the admirable steadiness with which he insisted upon stamping out hydrophobia, and also, notwithstanding the shrieks of the proprietors of mangy pets, upon resisting its re-introduction, has entitled him to the gratitude of his countrymen. His recent action, in the early establishment of an organisation adapted to deal with the absence of employment which is too often incidental to the winter season, is one equally entitled alike to the approval and to the active support of the public; and it is to be hoped that the arrangements for this purpose, of which he has laid the first stone in the metropolis, will before long be extended to all other parts of the country. The ordinary course of events, as our readers are likely only too well to remember, has been for the first slackness of work on the approach of winter to be seized upon by newspapers of a certain class, in search of what, in their peculiar modification of English, they would be pleased to call a "sensation," and to be described in moving epithets as an appalling condition of general "destitution" among worthy and respectable would-be workers. The immediate consequence has been the establishment of "relief funds" in various localities, funds usually administered by persons having neither the time nor the skill required for the proper sifting of the claims of applicants, and who, in the absence of these essential qualifications, have contentedly acted upon the principle that it is better to relieve a score of impostors than to suffer one deserving person to starve. They have failed, as a rule, to perceive the bearing upon their work of the injunction "these ought ye to have done and not to leave the other undone," or to recognise that the gift of money to a professional idler and beggar is, if not as bad as leaving the deserving to starve, at least a serious offence against the public weal. The committal of money to such hands has constantly been followed by a rapid and continuous immigration of the "work-shy" into the localities concerned; an immigration so continuous and so rapid as on several occasions to have brought about considerable increases of rent therein. The professionally "unemployed" and the professionally "distressed" found themselves quite able to pay freely for the privilege of residence where good things were going; and, in too many instances, the really unemployed and distressed were elbowed out to make room for their fictitious representatives.

Perhaps the first step in the direction of improvement was made by the Mansion House Committee which, last year, made arrangements for placing temporarily unemployed breadwinners upon farm colonies, where they were fed, housed, and employed upon agricultural work, their families being in the meanwhile supported at home. Only those men were taken who possessed established homes and who could show that they had been employed, all others being left to the operation of the Poor-law; and the results of the experiment, as published within the last few days, would appear to have been eminently successful. What Mr. Long proposes is, in fact, an extension of this scheme, to be carried into effect by the combined action of county or borough councils and boards of guardians, and to be so conducted as to check the large migration, ostensibly in search of "work," which now occurs into districts in which relief funds have either been established on a large scale or are administered in what the recipients would describe as a liberal manner. If this principle could be effectively carried out it would leave each district to deal with its own distress by the light of knowledge of the circumstances and characters of the applicants, and would at once put a stop to an enormous amount of imposture. Mr. Long suggests that the farm-colony system might be greatly extended, and it would presumably be possible, if such colonies on a large scale were established under sound management, to draft into them men coming from distances and paid for by the funds of their respective localities. A division into three classes is suggested as part of the scheme, according as the applicants may be decent workers of good character, or more or less casual in their industry, or irreclaimable loafers and idlers. It would be desirable, of course, that the three classes should be sent to different colonies, and Mr. Long expressed his willingness to seek for legislation which would permit men of the third class to be detained and made to work. There can, we think, be no doubt that the time has come for active measures in this direction, and especially for measures by which the indefinite increase and propagation of tramps and wastrels should be checked in the interests of the nation at large. Whatever virtues these people may possess, assuming, indeed, that they possess any, are of a kind incompatible with the requirements of modern civilisation; and their energies should now be directed into new channels. Separation of the sexes and compulsory labour for the adults, enforced by just so much of discipline or of punishment as might be necessary in order to secure the desired result, and the complete withdrawal of the children from the control and from the example of the parents, are urgently needed in the best interests, not only of the community, but of the tramps themselves. Whatever may be the intensity of their almost savage love for idleness and for freedom, their lives at the best must be extremely miserable, and their children at least might be guided to a desire for better things. In the meanwhile the whole class constitutes a dangerous nuisance and their influence in the diffusion of small-pox and other infectious diseases is alone a sufficient reason for the control of their wanderings by the operation of law.

